# Suicide in the Australian Mining Industry: Assessment of Rates among Male Workers Using 19 Years of Coronial Data

**DOI:** 10.1016/j.shaw.2023.03.003

**Published:** 2023-03-09

**Authors:** Tania King, Humaira Maheen, Yamna Taouk, Anthony D. LaMontagne

**Affiliations:** 1 Centre for Health Equity, Melbourne School of Population and Global Health, University of Melbourne, Victoria, Australia; 2 Institute for Health Transformation and School of Health and Social Development, Deakin University, Victoria, Australia

**Keywords:** Coronial data, Mining workers, Occupational mental health, Suicide

## Abstract

**Background:**

International evidence shows that mining workers are at greater risk of suicide than other workers; however, it is not known whether this applies to the Australian mining sector.

**Methods:**

Using data from the National Coronial Information System, rates of suicide among male mining workers were compared to those of three comparators: construction workers, mining and construction workers combined, and all other workers. Age-standardized suicide rates were calculated for 2001–2019 and across three intervals ‘2001–2006’, ‘2007–2011’, and ‘2012–2019’. Incidence rate ratios for suicide were calculated to compare incidence rates for mining workers, to those of the three comparative groups.

**Results:**

The suicide rate for male mining workers in Australia was estimated to be between 11 and 25 per 100,000 (likely closer to 25 per 100,000) over the period of 2001–2019. There was also evidence that the suicide rate among mining workers is increasing, and the suicide rate among mining workers for the period 2012–2019 was significantly higher than the other worker group.

**Conclusions:**

Based on available data, we tentatively deduce that suicide mortality among male mining workers is of concern. More information is needed on both industry and occupation of suicide decedents in order to better assess whether, and the extent to which, mining workers (and other industries and occupations) are at increased risk of suicide.

## 1 Introduction

In Australia in 2019, 3,318 Australians died by suicide [1]. Of these, 79% (2,626 individuals) were of working age (20–64 years) [1]. For the past decade, the proportion of suicides among the working age population has consistently remained at about 80% of all suicides in Australia. This has prompted interest in understanding occupational and workplace patterning of suicide, occupational risk factors and influences on suicide risk, and the viability of workplace settings for suicide prevention initiatives.

Some occupational groups and industrial sectors are known to be at higher risk of suicide than others. In the United States, recent research revealed that the group with the highest suicide rate was that of males in the mining, quarrying, oil and coal extraction industry, with a rate of 54.2 deaths/100,000, compared to 27.4/100,000 for the overall group of working males [2]. A 2013 study in the United Kingdom found that between the period of 2001 and 2005, coal miners had the highest suicide rate of all occupations examined, with a rate of 81 deaths per 100,000 worker years [3].

Evidence in Australia is less clear. A study in one state, Queensland, using data from the Queensland Suicide Register, found that employment in the mining industry was associated with lower suicide rates than employment in other industries [4]. This study, however, calculated crude suicide rates, not age-adjusted rates. Given that the age distribution of workers in the mining industry differs to that of the general working population, it is possible that this analytical approach underestimated the true rate of suicide among mining workers in Australia.

While little research has been carried out examining the suicide rate among Australian mining workers, some research has examined the mental health of mining workers. A study among Australian coal miners found higher levels of psychological distress than among a national sample of employed persons [5]. Higher levels of psychological distress among workers at remote mines have also been observed [[6], [7], [8]].

The extent to which occupational specific factors (e.g., type of work, exposures specific to particular occupations) contribute to differences in suicide risk and to what extent occupational variation in risk is related to compositional factors (e.g., gender, age distribution peculiar to different industries or occupations) is not clear. It is known that men are at much greater risk of suicide than women both in Australia [1] as well as internationally [13]. This in itself means that the workforce is at greater risk of suicide than other workforces that have a more equal gender distribution; however, the predominance of males in the mining industry also brings a culture that may heighten risk. Specifically, a culture of traditional masculine norms has been observed to predominate in mining settings [14], as well as in other male-dominated works settings [15]. It is known that certain masculine norms are associated with poorer mental health [16] and increased suicidal ideation [17,18]. Many mining workers are blue-collar, low-skilled workers, and in many settings, higher rates of suicide have been observed among lower-skilled and blue-collar workers both in Australia and internationally [19,20]. Of relevance, there is significant recognition of the fact that construction workers are at higher risk of suicide than the general working population in Australia [9,10], the United Kingdom [11,12], and the United States [2]. Given similarities between the construction and mining industries in terms of occupations (predominantly laborers and skilled trades workers), composition (male-dominated), and normative factors (prevalence of traditional masculine norms), it is possible that rates of suicide among mining workers are similar to that of construction workers.

In this study, we aimed to address the gap in understanding suicide among mining workers in Australia. Given that only 4% of suicides among those employed in mining are among women, investigation of deaths among female mining employees was not feasible, and for this reason, analysis was restricted to males. The research question guiding this analysis was: is there evidence that suicide rates among male workers in the Australian mining industry are elevated relative to other Australian male workers?

## 2 Methods

Drawing on coronial data from the National Coronial Information System (NCIS), a retrospective mortality design was adopted to examine specific suicide rates among male workers in the mining industry compared to other workers in Australia over time.

### 2.1 Dataset

The NCIS is a national repository containing mortality-specific information on deaths reported to coroners in Australia and New Zealand [21]. It contains data sourced from coronial briefs that are created as part of coronial investigations. Contained within the NCIS are coded and non-coded data, as well as searchable legal, medical, and scientific reports such as the coroner's findings, post-mortem, and toxicology reports and police summaries of death reports. Details regarding deaths are coded according to pre-specified fields by court-appointed staff in each state and territorial jurisdiction.

The NCIS database is a secure database and has a comprehensive search strategy that allows retrieving demographic information (such as age, sex, employment status, occupational text) and cause of death. As approved users of NCIS, study authors accessed the database and carried out the searches. The NCIS represents the best available information on suicide mortality in Australia and is used by various organizations and monitoring programs such as the Australian Bureau of Statistics, Safe Work Australia, and Australian Institute of Criminology [21]. A Quality Assurance Program has been developed to ensure the rigor and consistency of the data collection and coding program [22].

### 2.2 Inclusion criteria

Trained NCIS coders classify underlying causes of death using the International Classification of Diseases (ICD)-10 codes (X60-X84). Where deaths are deemed to be suicide, the ‘intent’ code is categorized as ‘intentional self-harm’. To be included in this study, cases were retrieved if intent type was intentional self-harm; the case was reported as closed; year of death was between 2001 and 2019; the person was employed at the time of death.

### 2.3 Ascertainment of industry/sector

Industry information about suicide cases was retrieved in two steps.

1. **Occupational text field**

In the NCIS database, each case has a ‘usual occupation’ text field which notes the person's occupation details at the time of death. This free-text field was searched for mining key words.

2. **Other free-text search**

NCIS web interface has a free-text search option to enable searches within police narratives of circumstances, coronial findings, toxicology, and autopsy reports. To ascertain industry-specific information where the occupational text was ambiguous, police and coroners' reports were used. Specifically, in some cases, coroners only report the broad occupation such as ‘laborers’, ‘engineers’ and may provide industry related information in their findings report, so the free-text field enabled us to identify additional mining workers that could not be identified definitively on the basis of occupation.

Appendix 1 provides a list of keywords used to search for relevant cases.

Cases identified by the two search strategies were merged, and duplicates were removed based on the unique NCIS identification codes.

**A note about industry and occupation**

The Australian Bureau of Statistics provides a classification structure for occupations (Australian and New Zealand Standard Classification of Occupation, ANZSCO) and industry/sector (Australian and New Zealand Standard Industry Classification, ANZSIC). One particular challenge is that common occupational titles can occur in multiple industries (e.g., a machinery operator might be working in the construction or mining industries).

Occupations are entered in the National Coronial Information System (NCIS) occupational field as free-text. In previous work, our team has coded the NCIS occupational text field according to ANZSCO [23]. That is, all deaths due to intentional self-harm from 2001 to 2019 have had their occupation at the time of death coded according to the ANZSCO four digit coding structure [23]. This information, however, was of limited value in identifying mining workers.

For this research, the ANZSIC classification is most relevant; however, information on industrial sector is only collected when the deceased was engaged in paid or unpaid work at the time of incident (that is, where the ‘activity’ category was classified as paid or unpaid work). Industrial sector information is not collected for cases of ‘intentional self-harm’ because the ‘incident activity’ is classified as ‘self-inflicted harm’—therefore in all suicide cases, industry information is not collected or coded by the NCIS.

To obtain relevant industry information, we needed to rely on the free-text fields (noted above). It is, therefore, the case that there is likely to be substantial misclassification of “industry”, with likely under-counting of mining workers (mining workers being misclassified as construction workers). As a consequence of this difficulty in distinguishing between mining and construction workers, we classified industry into four categories, defined below.

Given the difficulty in identifying mining workers as distinct from other workers (in particular, distinguishing mining from construction occupational titles/workers), we created four industry categories (see Table 1).

**Table 1:** Categorization of industry

#	Categorization	How category was derived
1	Mining	Open text fields: Occupation, Police reports, Coroners reports
2	Construction	ANZSCO coding (mining cases identified in classification 1 excluded)
3	Mining or construction	Combines category 1 and 2
4	Other occupations (neither mining nor construction). This served as the reference/comparison category.	Workers not in category 3

**Fig. 1:**
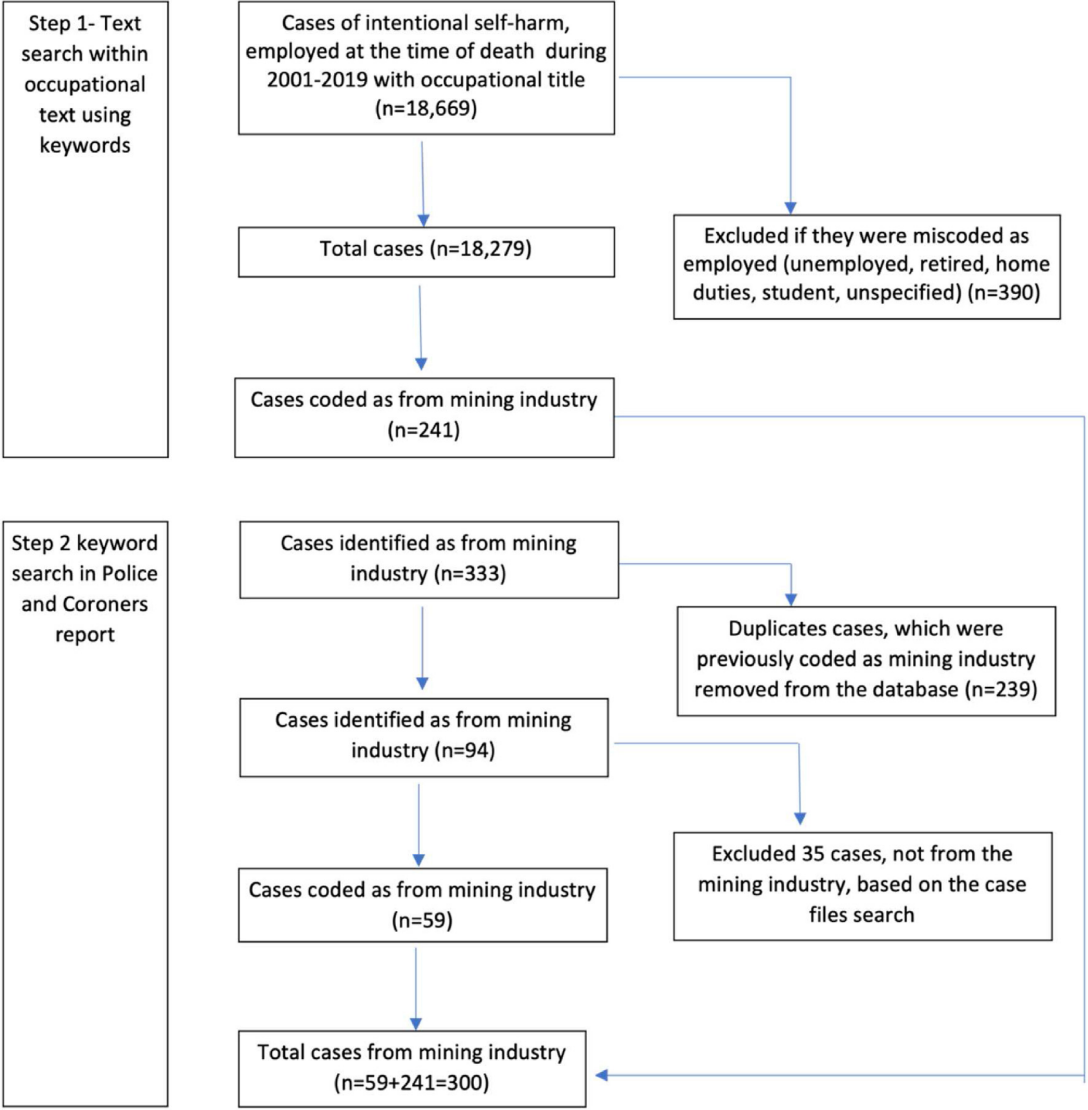
Identification of suicide cases employed in the mining industry.

Fig. 1 provides information regarding the search strategy.

### 2.4 Population estimates

Population estimates were obtained from the Australian Bureau of Statistics (ABS) using the 2006, 2011, and 2016 census data by industry, year, age, and sex. For 2001–2006, we used 2006 as the reference year; for 2007–2012, the reference year was 2011; for 2013–2019, we used 2016 as the reference year. The population data for mining workers were then adjusted annually by using quarterly labor force data (averaged for each year) from the ABS for mining workers. The adjustment accounts for the average change in population (each year) with reference to the corresponding census year. The Australian standard population (2001) from the ABS [24] was used to calculate age-standardized suicide rates.

### 2.5 Analytical approach

In descriptive analysis, we examined the age distribution of suicides for the different industry groups: mining workers, construction workers, mining and construction workers combined, and all other Australian workers. Frequencies and age-standardized suicide rates per 100,000 person-years pooled across time for each group were also calculated in all four industry groups.

The data were analyzed in year blocks due to sample size considerations—in particular, the mining category, our main category of interest, did not contain sufficient numbers to examine suicide rates yearly or in small time periods. Suicide rates for each industry group were, therefore, calculated across three intervals ‘2001–2006’, ‘2007–2011’ and ‘2012–2019’, with the choice of categories based on census years (2006, 2011, 2016). The number of suicide cases that occurred among workers in the mining industry each year was too small to analyze on a yearly basis. We calculated incident rate ratios (point estimate, 95% confidence interval) for construction, construction and mining, and mining workers compared to other workers (reference group) for the year groups; ‘2001–2006’, ‘2007–2011’, and ‘2012–2019’. All analysis was undertaken in Stata Version 16.0 [25].

## 3 Results

### 3.1 Age distribution of male suicides for each industry group

**Fig. 2:**
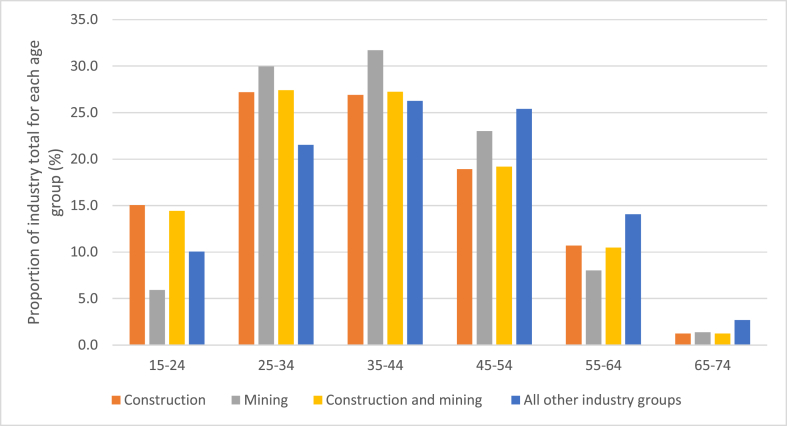
Age distribution of suicides among male mining and construction workers compared to Australian workers employed in other industries.

Fig. 2 shows the distribution of suicides (proportions) across year groups for male mining workers, construction workers, mining and construction workers, and all other workers. Among mining workers, the proportion of suicide deaths in 15–24-year-olds was lower than other industry groups. A higher proportion of mining suicides occurred in the 25–34- and 35–44-year age groups compared to other industry groups.

### 3.2 Age standardized suicide rates across industry

During the period between 2001 and 2019, 287 male workers employed in the mining industry died by suicide (Table 2). This corresponded to an age-standardized suicide rate of 11.4 per 100,000, similar to the rate calculated among males employed in industries outside of construction and mining (12.4 deaths per 100,000). The highest suicide rate was among construction workers (27.3 per 100,000 person years), among whom there were 3,819 deaths by suicide.

**Table 2:** Age-standardised suicide rates of male workers by industrial sector grouping

Industry	Number of suicide (2001–2019)	Worker years∗	Age-standardized suicide rate per 100,000 workers; 95% confidence interval
Mining	287	2,423,317	11.4 (9.7–13.1)
Construction (excluding mining)	3,819	13,341,401	27.3 (26.4–28.2)
Mining and construction (combined)	4,106	15,764,718	24.9 (24.1–25.7)
All other industry groups (neither mining nor construction)	11,069	85,475,756	12.4 (12.2–12.7)

∗ Total worker population across 2001–2019 (calculated on average worker population).

### 3.3 Time trends in age-standardized suicide rates for Australian male workers

**Fig. 3:**
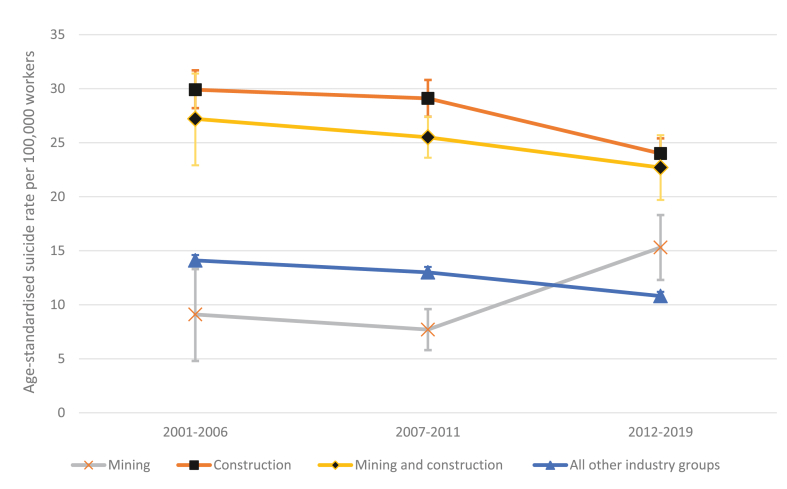
Age-standardized suicide rates between industry groups across three intervals.

Fig. 3 presents age-standardized suicide rates for male workers in the four industry groups across three 5–8 calendar-year intervals (based on the ABS census collection periods) between 2001 and 2019. Rates for all male workers employed outside construction and mining were relatively stable, measuring 14 deaths per 100,000 in 2001–2006, and declining to 11 per 100,000 in 2012–2019. The rates for construction workers appeared to be the highest of all industry groups across the three time periods, measuring 30 deaths per 100,000 in 2001–2006, and declining to 24 per 100,000 in 2012–2019: with the combined category of construction and mining workers slightly below this. The rates for both of these groups showed a decline in the third time period. This pattern contrasts with that of suicide deaths among workers in the mining industry, among whom rates were the lowest of all four industry categories in 2001–2006. For the period of 2007–2011, rates among the mining group declined (parallel with the other groups), but after this period, there was an increase in suicide deaths among mining workers for the period 2012–2019. This increase, occurring at the same time as a decrease in the other industry groups, is suggestive of convergence in suicide rates between mining workers and construction workers although additional years of data are needed to assess this trend definitively.

### 3.4 Incidence rate for suicide across industry categories

Table 3 shows the age-standardized incidence rate ratios (IRRs) for each of the industry categories relative to “all other workers” (employed males working in industries outside construction and mining). For the first period (2001–2006), the IRR for mining workers was significantly lower than “all other workers” (0.55, 95% confidence interval [CI] 0.40–0.74), while for construction workers, it was significantly higher (2.26, 95% CI 2.11–2.41). A very similar pattern was observed for the period of 2007–2011, with male construction workers dying by suicide at more than twice the rate of “other workers”, and mining workers dying by suicide at about half the rate of “other workers”. While the IRR for construction workers for the period of 2012–2019 remained consistent with that of previous years, the IRR for mining workers increased to 1.45, (95% CI 1.24–1.70), indicating that the suicide rate for mining workers was almost 1.5 times higher than that of other workers during that time.

**Table 3:** Incidence rate ratios (IRRs) male construction and mining workers compared to other workers, 2001 to 2019

Year block	Industry group	Total deaths by suicide	Worker years∗	Suicide rate	IRR	95% Confidence interval
2001–2006	All other workers	3,623	2,5099,578	14.1 (13.6–14.6)	1.00	Reference
	Mining workers	43	544,278	9.1 (4.9–13.4)	0.55	0.40–0.74
	Construction workers	1,198	3,676,800	29.9 (28.1, 31.6)	2.26	2.11–2.41
	Mining and construction workers	1,241	4,221,132	27.2 (25.6, 28.7)	2.04	1.91–2.17
2007–2011	All other workers	3,533	21,809,210	13.0 (12.5, 13.4)	1.00	Reference
	Mining workers	75	715,420	7.7 (5.8, 9.6)	0.65	0.51–0.81
	Construction workers	1,272	3,513,330	29.1 (27.4, 30.8)	2.24	2.10–2.39
	Mining and construction workers	1,347	4,228,750	25.5 (24.0, 27.0)	1.97	1.85–2.10
2012–2019	All other workers	3,913	39,608,528	10.8 (10.4–11.2)	1.00	Reference
	Mining workers	169	1,190,656	15.3 (12.3, 18.3)	1.45	1.24–1.70
	Construction workers	1349	6,328,496	24.0 (22.6, 25.4)	2.18	2.05–2.32
	Mining and construction workers	1,518	7,519,152	22.7 (21.4, 23.9)	2.07	1.94–2.21

∗ Total worker population across each year block (calculated on average worker population).

## 4 Discussion and conclusions

Using data from the NCIS, we examined suicide cases among Australian male mining workers for the period of 2001–2019. The suicide rate over time, examined in three time-blocks, suggested that while the rate for all other groups of male workers (construction workers, mining and construction workers together, all other workers) was declining, the rate among mining workers is increasing (2012–2019). The IRRs show that while the suicide rate for male mining workers in the period of 2012–2019 was significantly lower than that of male construction workers, it was significantly higher than it had been in the two previous time-blocks. Overall, while the suicide rate among male mining workers appeared to be lower than that of other male workers, we consider this to be an underestimate of the true suicide rate among mining workers—we expand upon this below.

Based on the available data, these results indicate that the best estimate of the true suicide rate among male mining workers is somewhere between 11 and 25 per 100,000. Given the similarities between the construction industry and the mining industry in terms of male-dominated composition and predominance of manual and trade occupations, it is reasonable to deduce that the estimate is closer to 25 than 11 per 100,000 (noting that it could also be outside these bounds).

Given classification challenges, our analysis also included a combined ‘construction and mining’ category which included all of those identified as either construction or mining workers. Given that construction workers outnumber mining workers by four to one, construction workers will predominate among occupational titles that could be in the mining or construction industries. This aside, it is clear that the combined group of mining and construction workers has a rate of suicide that is substantially greater than that of all other workers (those not employed in construction or mining).

The uncertainty of estimates makes it difficult to contextualize these results in the broader literature on suicide and mental health among mining workers. Our supposition that the suicide rate for male mining workers in Australia is at the upper end of the range between 11 and 25 per 100,000 aligns with other international work that has demonstrated elevated rates of suicide among mining workers. Such research has highlighted high rates of suicide among mining workers in the United States [2], as well as the United Kingdom [3]. Within Australia [9,10], and elsewhere such as the United Kingdom [11,12], and the United States [2], construction workers are known to be at greater risk of suicide than other workers, and the similarities between the construction and mining sectors adds strength to the supposition that the suicide rates for Australian male mining workers is at the upper end of this range.

The mining industry contributes substantially to Australian wealth. Together, mining services and resources account for 12% of the Australian gross domestic product [26] and 62% of Australia's total export revenue [27]. Annual mining production in Australia has experienced a significant boom period in the past 20 years [28], contributing substantially to Australian wealth, as well as the wealth of mining companies and owners. It is, therefore, incumbent on all of these parties, mining companies and owners and governments to evaluate and monitor suicide rates and act to prevent suicide among the workers who have produced and supported their wealth.

There are some unique factors that may increase suicide risk among mining workers. Mines are often located in remote townships or locations far from major cities. While many mining workers are resident, there has been increasing adoption of the fly in/fly out (FIFO) and drive in/drive out (DIDO) employment model in Australia [14]. Also commonly referred to as long distance commuting or ship in/ship out, this arrangement denotes workers who travel for work for a defined number of days (as per their roster) and then return home (often across long distances) for a break period. Where previously mining companies would construct a residential town for workers and their families, FIFO/DIDO arrangements have been increasingly embraced to the extent that in some states (Queensland and Western Australia), the proportion of employees living in large towns and commuting to mining towns in FIFO arrangements exceeds those living in the mining towns [14]. The increasing adoption of FIFO/DIDO models of working means that many mining workers are isolated from friends and family for long periods of time, and this remoteness and isolation, may increase risk of suicide for mining workers. Relatedly, it is possible that mining workers are exposed to psychosocial job stressors such as low job control and high job demands, which have been associated with increased risk of suicide [29]. It is also the case that occupational injuries may be a risk factor for suicides in the mining industry. Several studies have documented associations between occupational injury and suicide risk [[30], [31], [32]], with loss of income, depression related to the injury and disrupted family dynamics as being posited mechanisms for these associations [30]. It is also possible that the recent mining boom in Australia introduced stressors that precipitated increased suicide risk among workers. Future analysis could investigate these factors. Another avenue for future research is the investigation of suicide methods among mining workers, with comparison to other groups.

What this review has highlighted is the difficulty in reliably assessing suicide rates among mining workers, and by industrial sector more generally. Better data are needed. Ideally, detailed information on industry should be routinely collected in coronial data to enable the rigorous assessment of industry as well as occupational patterning of suicide. This would facilitate the identification of high-risk industries and occupations in general. As we note, the collection of occupational title information only is not sufficient for such analysis—industrial sector data are also needed. Importantly, workplace suicide prevention initiatives are commonly carried out at the industry level (such as within the construction industry or defense or mining), not at the occupational level (among specific occupations).

The most significant limitation of this report is the lack of industry data. This substantially limits confidence in the study findings and the inferences that can be made. As a consequence of this, it is highly likely that we have not identified all suicide cases in the mining industry. While there is relatively high certainty that those categorized in the mining category have been correctly classified—that is, they are miners and have been classified as miners—there is less certainty that all deaths among mining workers have been identified with the available data. More specifically, it is likely that many mining workers have been misclassified as construction workers because differentiating mining workers from construction workers is extremely difficult based on the occupational title data available. Many job titles are common to construction and mining (e.g., pipe fitter, machine operator) and have been coded by default as construction—where there is no (sector) distinguishing information in the rest of the decedent's record. If suicide occurs among miners at a higher rate than among the rest of the employed population, then such misclassification will likely bias the results towards the null (under-counting the total number of suicide deaths in the mining industry). It is therefore likely that the mining estimates derived in this analysis (11.4 per 100,000) may substantially underestimate the true suicide rate among mining workers. It is also possible that some decedents worked across multiple industries. Our analysis was unable to capture this, as only “usual occupation” of decedents was collected. This is not likely to be a major source of misclassification because on average in the Australian working population only 5–6% of working persons are multiple job holders [33]. There is some potential for bias due to confounding, as limited available covariate information meant that we were unable to control for confounding by factors such as education and income.

Another limitation of the NCIS data pertains to the length of time between death and case closure. Cases may remain open for a year or more, and as a consequence, suicide cases may be under-reported, particularly in more recent years. It is also the case that although the NCIS data are nationally standardized, each coronial jurisdiction is governed by its own Coronial Act. As a consequence, there are variations in data collection approaches and coding processes across state and territory jurisdictions, and this may contribute to some inconsistencies.

As a final point, it is often difficult to establish intent in suspected suicide cases, leading to undercounting of suicide cases [34]. It is estimated that suicide deaths are underestimated by 11–16% due to an inability to assess intent [34].

Despite these limitations, the NCIS dataset is the best available source for estimating suicide rates as it represents the most accurate and comprehensive information on suicide mortality that is available.

### Conclusion

In conclusion, it is difficult to confidently assess the extent to which mining workers are at elevated risk of suicide, given the significant data limitations. The likely misclassification of mining workers and the consequent under-estimation of mining suicide cases obfuscates assessment of suicide risk. Despite these limitations, there is some evidence that the suicide rate among male mining workers is increasing, and certainly, the rate among male mining workers for the period of 2012–2019 was significantly higher than that of “other workers” (excluding construction workers). This provides cautionary evidence that suicide mortality among mining workers may be of concern, particularly given the likely under-counting of suicides among mining workers. There is a clear imperative for more comprehensive industry and occupational information, and there is a need for advocacy to drive the collection of this important data.

## Funding

Partial funding for this work was received from MATES in Construction Australia and the Australian National Health & Medical Research Council (NHMRC) Partnership Project grant #APP1134499 and an NHMRC Future Fund Million Minds grant #MRF1199972. Humaira Maheen is a recipient of a Postdoctoral Fellowship funded by Suicide Prevention Australia. Tania King is supported by an Australian Research Council Discovery Early Career Research Award (DE200100607) funded by the Australian Government. Yamna Taouk is supported by a Victorian Health and Medical Research Fellowship funded by the Victorian Government.

## Ethics approval

This study was performed in line with the principles of the Declaration of Helsinki. Ethics approval was granted by the University of Melbourne Human Research Ethics Committee (#1748773.2), and the Coroners Court of Victoria Research Committee (CCOV RC 264) and the Justice Human Research Ethics Committee (JHREC).

## Data statement

Data are not publicly available but may be granted upon application from the National Coronial Information System.

## Conflict of interest

The authors declare no conflict of interest. Authors Associate Professor Tania King and Prof Anthony LaMontagne are current members of the (voluntary, unpaid) National Research Reference Group for Mates in Construction, and Prof LaMontagne is a (voluntary, unpaid) Director on the MATES in Construction Board. Dr Humaira Maheen, and Dr Yamna Taouk do not have non-financial interests.
