# Supplementary material for: Suicide in the Australian Mining Industry: Assessment of Rates among Male Workers Using 19 Years of Coronial Data
Source: Saf Health Work. 2023 Mar 9;14(2):193–200. doi: 10.1016/j.shaw.2023.03.003 (PMC10300480; doi:10.1016/j.shaw.2023.03.003)
Supplement: Multimedia component 1 [file mmc1.docx]

# APPENDICES

## Appendix 1: Keywords used to search free text fields to identify mining workers

1. Mine
2. Mining
3. Miner
4. Mineworker
5. Coal
6. Iron
7. Gas
8. Gold
9. Minerals
10. Resources
11. FIFO/DIDO
12. Ore
13. Oil
14. Metals
15. Quarry
16. Petroleum
17. Silver
18. Materials
